# Case Report: A novel use of Transcranial Pulse Stimulation in refractory Restless Legs Syndrome

**DOI:** 10.3389/fnhum.2026.1723992

**Published:** 2026-02-25

**Authors:** Ellie Mitsi, Petros Kattou, Sergey Kondratev

**Affiliations:** Research Department, SOZO Brain Center, Nicosia, Cyprus

**Keywords:** brain stimulation, case report, Functional Network-Oriented Neuromodulation, non-invasive brain stimulation, Restless Legs Syndrome, Transcranial Pulse Stimulation

## Abstract

**Introduction:**

Restless Legs Syndrome (RLS) is a sensorimotor disorder characterized by an irresistible urge to move the legs. When resistant to pharmacological and psychotherapeutic treatments, RLS remains highly debilitating. This case study represents the first documented case of Transcranial Pulse Stimulation (TPS) applied in treatment-resistant RLS, exploring its feasibility, tolerability, and potential clinical effects.

**Methods:**

A 56-year-old male diagnosed with RLS with severe, persistent bilateral leg restlessness and neuropathic cramping pain underwent six sessions of TPS target cortical and subcortical network hubs within the Functional Network-Oriented Neuromodulation (FNON) framework.

**Results:**

At six-week follow-up, the patient reported improved sleep, a 95.2% reduction in RLS severity, a 66.7% decrease in pain/discomfort, and a 25% improvement in overall health status. Depression/Anxiety symptoms decreased by 25% while mobility and self-care remained stable at normal levels.

**Conclusion:**

In this single case, TPS was well tolerated and associated with meaningful symptomatic improvement. While no conclusions regarding safety or efficacy can be drawn from an individual observation, these findings suggest that TPS may warrant further investigation as non-invasive neuromodulatory approach for refractory RLS. This report expands the scientific literature by introducing a novel network-based neuromodulation modality to the RLS field and provides a foundation for future controlled studies to validate efficacy and optimize stimulation protocols.

## 1 Introduction

RLS is a sensorimotor disorder characterized by an irresistible urge to move the legs (Gossard et al., 2021). Thus, it results to an unpleasant crawling or throbbing sensation in the feet, calves and thighs, which worsens during the night (Xu et al., 2025). RLS is extremely incapacitating when resistant to conventional treatments, including pharmacological and physiotherapeutic interventions, leading to significant challenges in management. While neuromodulation approaches have demonstrated promise in a number of refractory neurological and psychiatric disorders, there is no evidence about its effectiveness in RLS (Silber et al., 2021). Transcranial Pulse Stimulation (TPS) is a non-invasive neuromodulation therapy that delivers short, repetitive shockwaves through a neuro-navigated device (Ngan and Cheng, 2025). Since its first introduction in 2019 in Alzheimer’s disease patients (Beisteiner et al., 2020), TPS has shown promising effects in several conditions, including Parkinson’s Disease (Osou et al., 2024), Autism (Cheung et al., 2023), Attention deficit hyperactivity disorder (ADHD) (Cheung et al., 2024) and Depression (Cheung et al., 2023). Results from studies suggest that the delivered ultra-sonic pulses result to a wide range of VEGF (Vascular Edonthelial Growth Factor), metabolic and neuroangiogenesis (Cont et al., 2022). In addition, Extracorporeal Shock Wave Therapy (ESWT) promotes biological and neurological effects through a combination of mechanical conduction, angiogenesis, vacuolation and biochemical signals (Yang et al., 2024). The FNON model, clinically applied at the SOZO Brain Center, provides a conceptual framework for understanding how neurological disorders, including RLS, arise from dysfunctional integration across large-scale brain networks rather than isolated regions. TPS fits well within the FNON paradigm because it allows for the precise stimulation of multiple interconnected cortical and subcortical hubs to restore large-scale network connectivity.

We present the case of a 56-year-old male with severe, persistent, bilateral leg restlessness and neuropathic cramping pain extending from the thighs to the knees. Given the refractory nature of the disease and the absence of effective symptom control, we applied TPS within the FNON framework to explore its feasibility and potential clinical effects in this patient. This case is unique at it represents the first documented application of TPS in treatment-resistant RLS, integrating the FNON network-based approach to target multisystem dysfunction. It provides preliminary, hypothesis-generating evidence that TPS may influence sensory-motor-limbic network activity and supports further systematic investigation of this approach in refractory RLS.

## 2 Case description

### 2.1 Case presentation

**Figure 1:**
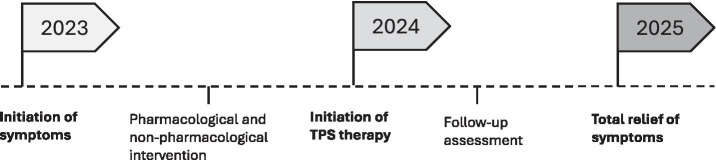
Timeline of clinical course and interventions in a patient with treatment-resistant RLS treated with TPS.

The patient was a 56-year-old Caucasian male who presented with a history of severe, persistent, bilateral leg restlessness accompanied by neuropathic cramping pain extending from the thighs to the knees. Symptoms began in 2023 (Figure 1) and were most pronounced at night, leading to frequent awakenings, poor sleep, and daytime fatigue. He reported a constant urge to move his legs to relieve discomfort. No family history of RLS was reported. He had previously undergone multiple therapeutic interventions. Pharmacological treatments included dopaminergic agents and gabapentinoids, which were either ineffective or produced only transient relief. Non-pharmacological strategies such as physiotherapy, acupuncture, and electrical stimulation modalities were also attempted but did not result in sustained benefit. The persistence of symptoms despite multiple appropriate therapeutic interventions indicated a refractory course of RLS.

### 2.2 Clinical assessment

A comprehensive neurological and physical examination was performed prior to treatment to establish the patient’s baseline clinical status. The assessment included evaluation of motor strength, tone, coordination, reflexes, sensation, and gait to identify any focal neurological deficits. Deep tendon reflexes, balance, and proprioception were examined to rule out peripheral neuropathy or central nervous system involvement. The presence, distribution, and intensity of restlessness, cramping, and discomfort in the lower limbs were also assessed through direct clinical observation and patient reporting.

Two validated assessment tools, the Restless Legs Syndrome Severity Scale (RLS-SS) and the EuroQol 5-Dimension 5-Level Questionnaire (EQ-5D-5L), were used to evaluate the impact of TPS in this patient. On a 5-point scale, the EQ-5D-5L assesses five health-related domains: mobility, self-care, daily activities, pain/discomfort, and anxiety/depression, with lower scores indicating better health. The patient’s self-rated overall health status was additionally recorded using the EQ-5D Visual Analogue Scale (EQ-5D-5L-VAS), ranging from 0 (worst imaginable health) to 10 (best imaginable health). The RLS-SS consists of 10 items scored from 0 to 4, yielding a total score ranging from 0 to 40, with higher scores indicating greater symptom severity. Based on established clinical cut-offs, scores of 0–10 indicate mild RLS, 11-20 moderate RLS, 21-30 severe RLS and 31-40 very severe RLS. Both assessments were conducted at baseline (pre-treatment) and at follow-up in 6 weeks (intra-treatment phase), allowing for a direct comparison of changes in quality of life and RLS symptom burden.

### 2.3 TPS parameters

**Figure 2:**
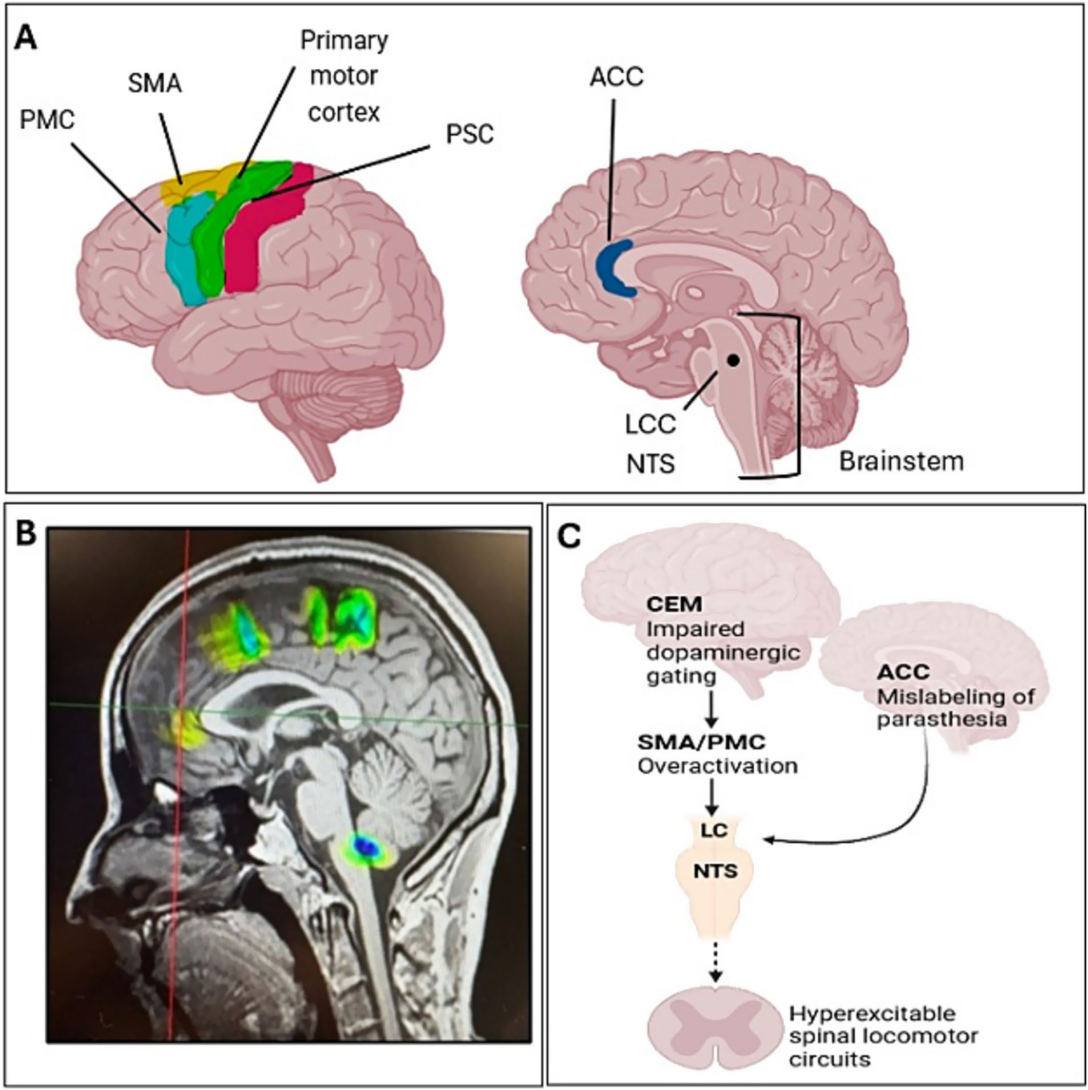
Multimodal illustration of network dysfunction and neuromodulation targeting in RLS. **(A)** Upper right: lateral schematic view of cortical regions implicated in RLS within the FNON framework: premotor cortex (PMC, yellow), supplementary motor area (SMA, green), primary motor cortex (M1, red), and primary somatosensory cortex (PSC, blue). Upper left: Sagittal schematic highlighting subcortical and brainstem hubs: anterior cingulate cortex (ACC, deep blue), locus coeruleus (LC), nucleus tractus solitarius (NTS). **(B)** Control MRI showing TPS targeting of cortical and brainstem hubs, with green activation clusters corresponding to stimulation foci. **(C)** Integrative schematic of the FNON model in RLS: impaired dopaminergic gating by the central executive mode (CEM) drives SMA/PMC overactivation, PSC amplification of paresthesia, and ACC mislabeling of salience. Dysfunctional coordination of LC and NTS weakens descending inhibition, resulting in hyperexcitable spinal locomotor circuits (created from Biorender.com).

A TPS trial using the Neurolith device by Storz Medical, was proposed as a novel neuromodulation intervention in RLS due to the refractory nature of the disease. Since brain MRI from the patient was unavailable, a control MRI of a healthy individual was used for TPS neuro-navigation and targeting. The patient received a full course of TPS treatment targeting the Supplementary Motor Area (SMA), Premotor Cortex (PMC), Primary Somatosensory Cortex (PSC), the Anterior Cingulate Cortex (ACC) and the Brainstem (Figure 2). Stimulation was delivered sequentially to each target region, with all predefined brain areas stimulated during every TPS session. A total number of 6 in-clinic sessions were performed over a period of 3 weeks. Stimulation parameters included 6,000 pulses per session at an energy flux density of 0.25 mJ/mm^2^ and a pulse frequency of 4 Hz. No anesthesia or sedation was required, and no adverse effects were reported during or after any session.

## 3 Results

At baseline, the neurological examination revealed no focal motor or sensory deficits, with normal muscle strength, tone and coordination. Deep tendon reflexes were preserved and symmetrical, and gait and balance were unaffected, confirming the absence of major motor impairment.

By the sixth week the patient reported a significant improvement in symptoms. He described improved sleep continuity, reduced nocturnal discomfort, and a decreased urge to move the legs. No new neurological deficits or side effects were observed during or after the sessions, and no additional medications or interventions were introduced during the treatment period.

**Figure 3:**
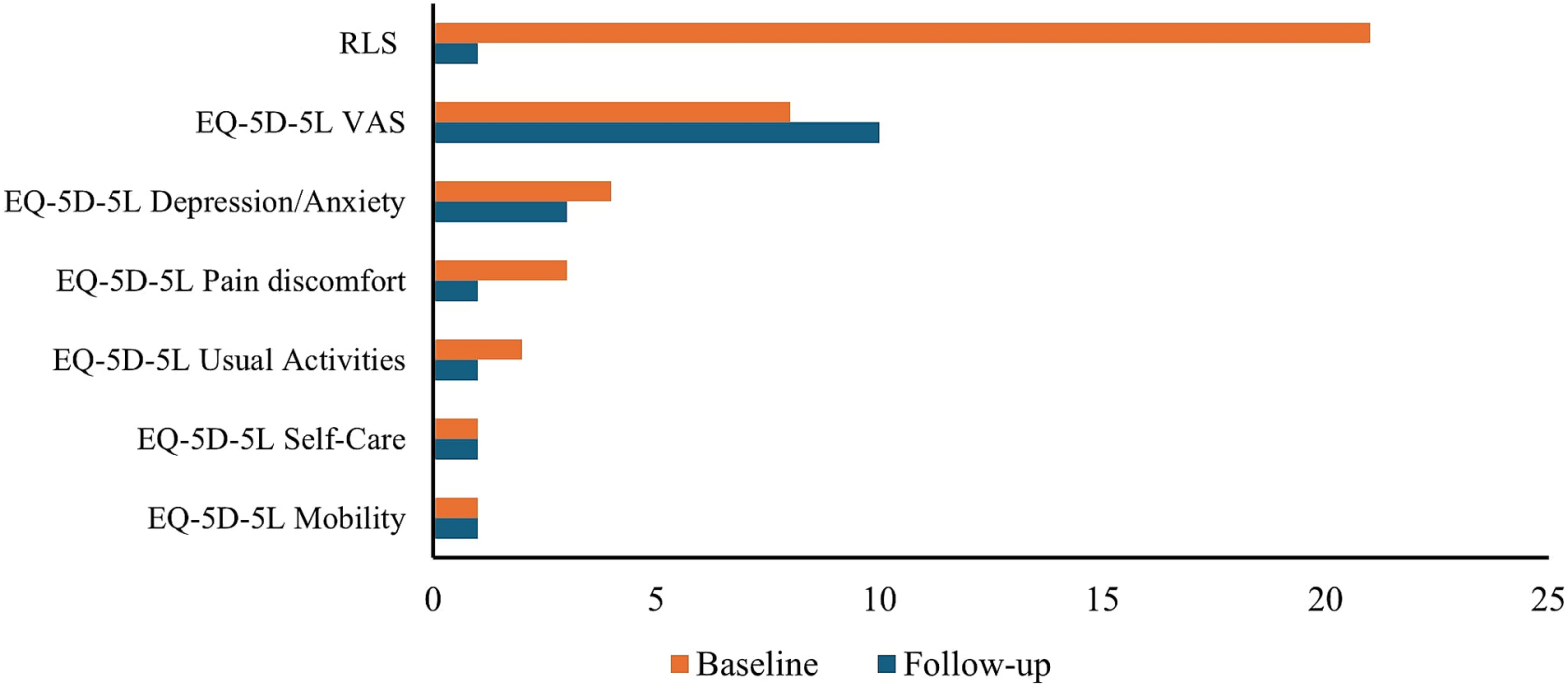
Baseline and follow-up scores across EQ-5D-5L domains and RLS severity following TPS. The bar chart compares baseline and follow-up scores across EQ-5D-5L domains and RLS severity after TPS. Results show a marked reduction in both RLS severity and improvements in health-related quality of life.

Quantitative assessments performed 2 weeks after the last TPS session confirmed these improvements (Table 1). Compared with the baseline, there was a 50% decrease in daily activities impairment, a 66.7% reduction in pain/discomfort, a 25% reduction in depression/anxiety symptoms, and a significant 95.2% reduction in RLS severity scores. In addition, the EQ-5D-5L VAS score improved by 25%, indicating better health status. Notably, mobility and self-care scores remained stable, consistent with their baseline normal status. These results are illustrated in Figure 3.

**Table 1:** Percentage change between baseline and follow-up assessments following TPS.

Variable	Baseline	Follow-up	Improvement (%)
EQ-5D-5L mobility	1	1	0
EQ-5D-5L self-care	1	1	0
EQ-5D-5L daily activities	2	1	50
EQ-5D-5L pain/discomfort	3	1	66.7
EQ-5D-5L depression/anxiety	4	3	25
EQ-5D-5L VAS	8	10	25
RLS	21	1	95.2

For EQ-5D-5L dimensions and RLS, lower scores indicate improvement. For VASs, higher scores indicate improvement.

## 4 Discussion

This case report demonstrates the potential feasibility and exploratory clinical effects of TPS as a non-invasive neuromodulation approach for treatment-resistant RLS using the FNON approach. Unlike conventional therapies that often focus on neurotransmitter modulation, TPS acts through mechanical stimulation of interconnected cortical and subcortical hubs, restoring network-level balance. The clinical improvement observed after TPS supports the importance of targeting dysfunctional circuits rather than isolated brain regions.

Within the FNON framework, RLS may reflect dysfunction of large-scale networks spanning sensorimotor, limbic, and descending modulatory circuits. Altered connectivity involving the SMA has been documented in RLS. For instance, increased functional coupling between a brainstem cluster and SMA was observed in RLS patients and correlated with symptom severity (Xu et al., 2020). Additional, structural studies also reveal cortical changes in somatosensory regions, such as reduced thickness in the postcentral gyrus, implicating the PSC in the sensory component of RLS pathophysiology (Rizzo et al., 2017). Together, these findings might indicate that overactivation of SMA/PMC circuits, amplification of sensory input via PSC, and aberrant salience processing in ACC may interact to reinforce the urge to move behaviors that is a key characteristic in RLS.

Besides the cortical networks, the brainstem also plays an important role. Gray matter density and functional connectivity changes in the pons and midbrain regions have been reported in RLS, suggesting that subcortical and brainstem circuits may contribute to symptom generation (Xu et al., 2020). The modulation of descending pathways; noradrenergic (LC) and visceral/vagal (NTS), is well established in neurophysiology, and their dysregulation could possibly predispose to spinal hyperexcitability, a phenomenon documented in RLS via altered reflex responses. Even though evidence implicating LC or NTS in RLS is limited, neuromodulation studies provide mechanistic plausibility. For example, TPS has been shown to influence deeper structures and network connectivity in other neurological disorders, supporting the hypothesis that it may rebalance descending modulatory tone (Osou et al., 2024; Cont et al., 2022). By simultaneously stimulating these hubs, TPS may recalibrate sensory, motor, and limbic network interactions, producing a synergistic therapeutic effect. For example, the PSC is crucial for processing somatic sensations and has been associated with abnormal sensory experiences of RLS patients (Dinse et al., 2023). Moreover, abnormalities in the Central Executive Mode (CEM) have also been reported in patients experiencing fatigue, sleep disturbances, and emotional distress (Bigliassi et al., 2025). Therefore, targeting the CEM might result in the reduction of cognitive and affective burden of RLS by modulating large-scale network connectivity. In addition, the brainstem contains key nuclei involved in motor control, pain processing, and arousal, all of which can be altered in RLS pathology (Godau et al., 2008).

While the precise mechanisms remain unclear, the gradual symptom reduction observed across sessions supports the hypothesis of neuroplastic adaptation rather than acute suppression. These results suggest a possible disease-modifying effect, rather than temporary symptomatic relief. Importantly, the absence of side effects reinforces the feasibility of TPS, particularly for patients who are unresponsive to or intolerant of pharmacological treatments.

The main strength of this report lies in its novelty, as it is the first clinical application of TPS in RLS and the first to demonstrate symptom relief via multimodal cortical targeting guided by FNON principles. The use of FNON framework represents a conceptual advancement, emphasizing a network-based therapeutic rationale rather than isolated regional targeting. This perspective aligns with emerging models of RLS as a multisystem disorder involving sensory, motor, and limbic dysregulation. An additional strength of this study is the comprehensive clinical assessment that included validated outcome measures, a detailed neurological examination, and systematic follow-up to capture both subjective and quantitative improvements. The absence of adverse effects across multiple treatment sessions further supports the tolerability of TPS, consistent with findings from its use in other neurological disorders. However, several limitations must be acknowledged. Foremost, single-case design limits the generalizability of the findings and precludes definitive conclusions regarding efficacy or safety. Additionally, neuronavigation was performed using a standardized MRI from a healthy control rather than patient-specific imaging. While this may have reduced targeting precision, it did not prevent the observed clinical benefit.

In summary, this case adds to the emerging body of literature supporting network-targeted neuromodulation for RLS. By demonstrating the feasibility and tolerability of TPS in a treatment-resistant patient, and its association with symptom improvement, this report provides preliminary evidence for a novel, non-invasive therapeutic approach. These findings warrant systematic investigation in controlled clinical studies to evaluate safety, efficacy, and the potential for network-level modulation in RLS.

## Patient perspective

“For the first time in months, I could sleep through most of the night without the constant need to move my legs. The therapy was comfortable, and I didn’t feel any side effects”.

## Data Availability

The datasets presented in this study can be found in online repositories. The names of the repository/repositories and accession number(s) can be found in the article/supplementary material.
